# Applying machine learning to pharmacovigilance data: A proof‐of‐concept study

**DOI:** 10.1002/bcp.70655

**Published:** 2026-06-17

**Authors:** Romain Barus, Pauline Schiro, Jean‐Luc Faillie, Romane Freppel, Johana Béné, Claire de Canecaude, François Montastruc

**Affiliations:** ^1^ Department of Medical and Clinical Pharmacology, Centre of PharmacoVigilance and Pharmacoepidemiology, Faculty of Medicine Toulouse University Hospital Toulouse France; ^2^ Department of Medical Pharmacology and Toxicology IDESP, Univ Montpellier, INSERM, CHU Montpellier Montpellier France; ^3^ Centre Régional de Pharmacovigilance Université Bourgogne Europe, CHU Dijon Bourgogne Dijon France; ^4^ Regional Pharmacovigilance Center, Department of Clinical Pharmacology Lille University Hospital Lille France

**Keywords:** artificial intelligence, adverse reactions, pharmacovigilance, machine learning, w2rfarin

## Abstract

**Aim:**

Machine learning (ML) applications in pharmacovigilance remain limited and underexplored. Using data from the French National pharmacovigilance database (FNPV), this proof‐of‐concept study aimed to assess the feasibility of using a ML algorithm—eXtreme Gradient Boosting (XGBoost)—combined with SHapley Additive exPlanations (SHAP) analysis, to classify a well‐defined drug–adverse reaction pair (warfarin‐associated gastrointestinal bleeding) and identify risk factors for reporting this adverse reaction.

**Methods:**

We extracted individual case safety reports (ICSRs) involving warfarin from the FNPV. Following data preprocessing, the dataset was randomly split into a training set (75%) and an independent test set (25%). The XGBoost algorithm was trained to classify reports as either gastrointestinal bleeding or non‐gastrointestinal bleeding adverse reactions. Model performance was assessed on the test set using multiple metrics: accuracy, recall, precision, F1‐score and the area under the receiver operating characteristic curve (AUC‐ROC). Feature importance and directional effects were interpreted using SHAP values.

**Results:**

A total of 2025 ICSRs involving warfarin were extracted from the FNPV. After preprocessing, 1045 ICSRs were retained. On the test set, the XGBoost model demonstrated an accuracy of 0.68, recall of 0.80, precision of 0.68, F1‐score of 0.74 and an AUC‐ROC of 0.716. SHAP analysis revealed the four most influential features in predicting gastrointestinal bleeding reporting: age, gastrointestinal bleeding risk, antiplatelet and history of renal dysfunction.

**Conclusion:**

We demonstrated the feasibility of using an XGBoost algorithm combined with SHAP analysis to pharmacovigilance data for the classification of adverse drug reactions and identify predictive features of reporting, following post hoc model interpretation by pharmacologists.

**What is already known about this subject**

- Machine learning (ML) is used in specific clinical pharmacology areas like therapeutic drug monitoring.
- In pharmacovigilance, the feasibility of ML is not yet well‐established.

**What this study adds**

- We demonstrate that ML (eXtreme Gradient Boosting model) can be used on pharmacovigilance data to classify adverse drug reactions of interest.
- ML requires post hoc model interpretability by pharmacology experts to identify potential predictive features of adverse drug reactions reporting.

## 1 INTRODUCTION

Artificial intelligence (AI) is gaining attention in pharmacovigilance. The assistance of generative AI to support pharmacologists has been evaluated in drug monitoring service activities,^1^, ^2^ in pharmacovigilance case documentation^3^ and literature screening.^4^ However, relatively few studies have specifically investigated the application of machine learning (ML) methods in pharmacovigilance.^5^, ^6^, ^7^

ML is a subset of artificial intelligence that involves the development of algorithms capable of learning patterns from data without being explicitly programmed. These models use statistical techniques to make predictions or classifications based on input features.^8^

In clinical settings, ML has already demonstrated significant utility. For instance, ML has been applied in therapeutic drug monitoring to predict drug concentrations, especially for medications with a narrow therapeutic index, using regression‐based approaches.^9^ In nephrology, ML models have been employed to classify and predict outcomes such as kidney graft rejection.^10^ Despite these benefits, one of the main criticisms of ML has historically been its ‘black box’ nature, the lack of transparency in how decisions or predictions are made, which limits its acceptance in high‐stakes domains like healthcare.^7^ To address this limitation, recent advances in explainable AI have introduced tools that enhance the interpretability of complex models. Among these, the SHapley Additive exPlanations (SHAP) framework has emerged as a powerful method for explaining individual predictions. SHAP values provide insight into which features contribute most to a model's output, thereby increasing transparency in ML‐based decision‐making processes.^6^

In pharmacovigilance, ML approaches hold particular promise for predicting the likelihood of individual adverse drug reactions (ADRs).^7^ By analysing large datasets like pharmacovigilance datasets, ML models could identify subtle patterns that may be missed by traditional methods in a hypothesis‐free manner. To date, one well‐conducted publication has tested ML for identifying ADR risk factors reporting in a pharmacovigilance database. Attelin et al. applied a random forest algorithm combined with Boruta feature selection to the Swedish national pharmacovigilance database to identify risk factors for bleeding events in patients taking direct oral anticoagulants.^5^ This study did not report performance metrics, limiting the assessment of the ML model's utility and robustness.

To address this gap, we conducted a proof‐of‐concept study focusing on a well‐defined drug–adverse reaction pair: warfarin and gastrointestinal (GI) disorders. Risk factors for warfarin‐associated bleeding are well‐established,^11^, ^12^ among them, age and renal failure are associated with a higher risk of bleeding. Using French pharmacovigilance data, we aimed to evaluate the feasibility of applying a robust ML algorithm—eXtreme Gradient Boosting (XGBoost)—in combination with SHAP analysis to classify GI bleeding cases associated with warfarin use and rank known risk factors of bleeding.

## 2 METHODS

### 2.1 Datasource

We used the French National PharmacoVigilance database (FNPV) to extract all individual case safety reports (ICSRs) registered since the database creation (1985) and involving warfarin on 4th of May 2025. In the FNPV, before being recorded, each ICSR has been rigorously evaluated by a pharmacologist specialized in pharmacovigilance to perform a causality assessment. ADRs are coded according to the Medical Dictionary for Regulatory Activities (MedDRA).

### 2.2 Reports (ICSRs) selection

We selected ICSRs in which warfarin was coded as suspect in the occurrence of a gastrointestinal bleeding or any other ADR not involving bleeding. ICSRs of GI bleeding were identified by using the following narrow Standardized MedDRA Queries (SMQs): gastrointestinal haemorrhage. ICSRs not involving bleeding (any other ADRs not involving any sort of bleeding) were selected by excluding all the ICSRs that contained the following SMQs terms: Gastrointestinal haemorrhage (SMQ); Central nervous system haemorrhages and cerebrovascular conditions; haemorrhage laboratory terms (SMQ); haemorrhage terms (excl laboratory terms) (SMQ); conditions associated with central nervous system haemorrhages and cerebrovascular accidents (SMQ); haemorrhages (SMQ); haemorrhagic central nervous system vascular conditions (SMQ); gastrointestinal perforation, ulcer, haemorrhage, obstruction non‐specific findings/procedures (SMQ) and gastrointestinal perforation, ulceration, haemorrhage or obstruction (SMQ).

Finally, all ICSRs were categorized in two groups: GI bleeding or other ADR not including bleeding. When an ICSR included multiple ADR and at least one of these was a GI bleeding, the report was included in the GI bleeding group.

### 2.3 Data preprocessing

Data preprocessing was conducted to ensure consistency, remove irrelevant or erroneous ICSRs and creating features essential for analysis. All operations were performed using R (R version 4.4.2) and relevant packages including dplyr, stringr and skimr.

ICSRs involving foetuses, newborns and cases occurring in patients under 18 years of age were excluded. Warfarin doses were harmonized. When dose units were listed as ‘dosage form’, numeric values were extracted from medication names using regular expressions. Dosage frequencies were also accounted allowing for the calculation of a normalized daily dose expressed in milligram. ICSRs containing aberrant data regarding doses were also excluded. ICSRs involving overdosage without any adverse event were also excluded.

Features were derived from suspected or concomitant medications, including use of anticoagulants, antiplatelets, nonsteroidal anti‐inflammatory drugs (NSAIDs), serotonin reuptake inhibitors (SRI), liver enzyme inducers, valproate, cephalosporins and proton pump inhibitors. The list of concomitant medications is available in Table S1. These medications were selected as we hypothesized that they could increase the risk of GI bleeding either by themselves or by interacting with warfarin. Based on patient's history (extracted with MedDRA High Level Group Term, HGLT), the following features were also created: gastrointestinal bleeding risk factor, renal dysfunction history and liver disease history. A gastrointestinal bleeding risk factor was coded if the patient's history included any of the following HLGT: gastrointestinal ulceration and perforation, gastrointestinal haemorrhages NEC, malignant and unspecified neoplasms gastrointestinal NEC, gastrointestinal vascular conditions (HLGT) or gastrointestinal inflammatory conditions. These features were chosen based on previously described clinical risk factors for bleeding.^13^ A liver disease history was coded if the patient's history contained Hepatic and hepatobiliary disorders (HLGT). A renal dysfunction history was coded if renal disorders (excluding nephropathies) (HLGT) was present in the patient's history. ICSRs with missing data on dosage, sex and age were excluded. If alcohol or tobacco use was not reported, the patient was coded by default as non‐alcoholic or non‐smoker. The binary outcome feature, *GI_bleeding*, was encoded as a factor with two levels (‘0’, ‘1’). Finally, stratified data partitioning was performed using the initial_split() function from the rsample package. The split ratio was 75% for training and 25% for testing.

### 2.4 Development and interpretability of XGBoost model

We developed a predictive model for GI bleeding associated with warfarin using an XGBoost algorithm in R studio 2024.09.1 by using R (version 4.4.2). The supervised classification pipeline was implemented using the tidymodels framework in R. All predictor variables were included in a preprocessing workflow defined with recipe(). Model training was performed via the xgboost engine. To identify the optimal hyperparameters, we used fivefold stratified cross‐validation with the vfold_cv() function, ensuring the class distribution of the target variable (GI_bleeding) was preserved across all folds. Hyperparameter tuning was conducted using a Latin hypercube sampling strategy (grid_latin_hypercube()) to generate a space‐filling grid of 20 parameter combinations. Grid search was carried out using tune_grid(), with resampling control settings configured to retain predictions and workflows. The selection of the best hyperparameter set was based on maximizing the mean Area Under the ROC Curve (ROC AUC) across resampled folds. The final set of hyperparameters was as follows:

trees = 1090.

tree_depth = 6.

learn_rate = 0.01769449.

loss_reduction = 3.115073e‐06.

sample_size = 0.4992591.

mtry = 2.

min_n = 9.

The final model was then retrained using xgboost::xgb.train() on the entire training dataset with the selected hyperparameters. Feature importance was assessed using the vip::vip() function.

Model performance was evaluated on the test dataset using the following metrics: ROC AUC, accuracy, sensitivity (recall), precision, F1‐score and the confusion matrix. Additionally, ROC curve was plotted using the PRROC package. The whole process is described in Figure 1.

**FIGURE 1:**
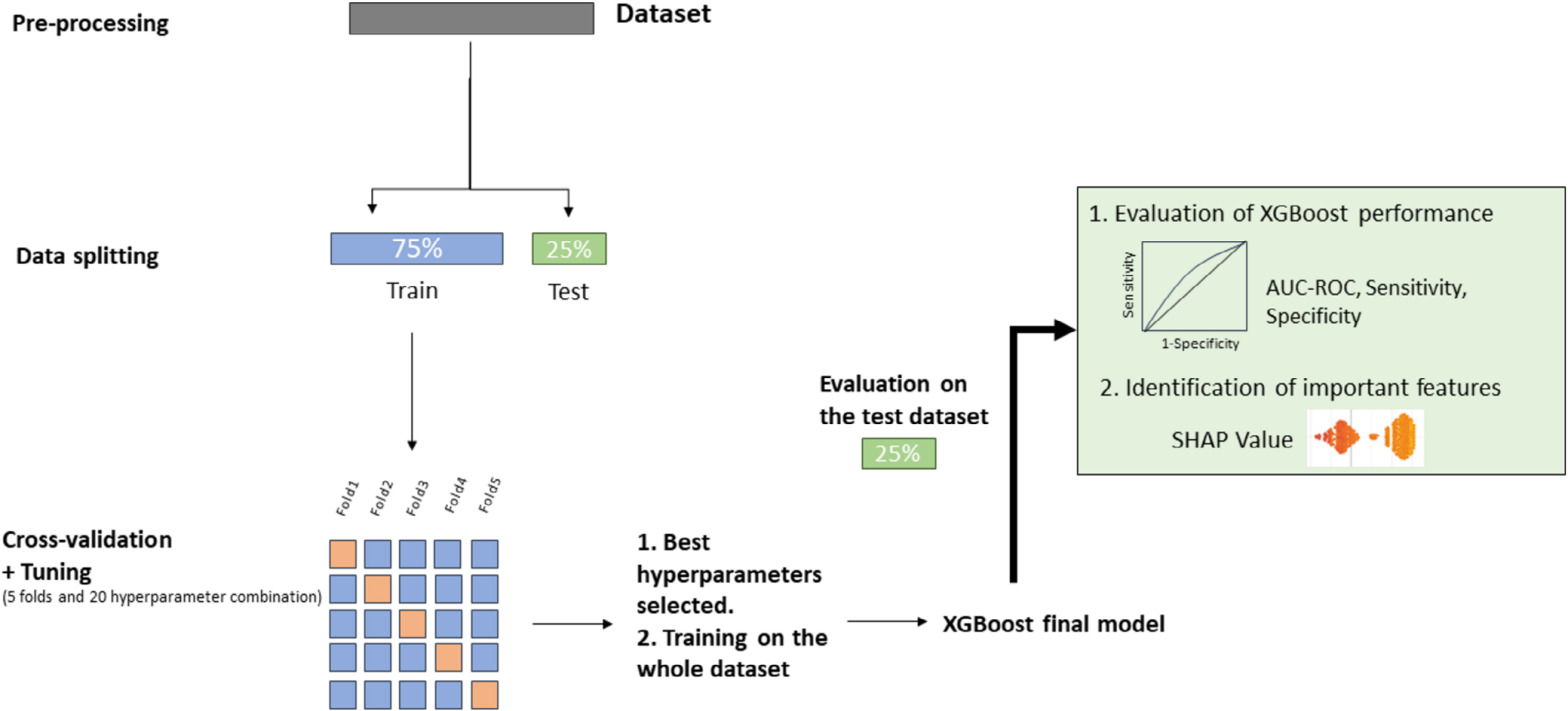
Machine learning workflow. Preprocessing: the dataset is split into a training set (75%) and a test set (25%). After preprocessing, an XGBoost model is developed using fivefold cross‐validation with tuning across 20 hyperparameter combinations. The best hyperparameters are then selected to train the final model on the full train dataset. Model performance is evaluated on the test set using area under the ROC curve (AUC‐ROC), sensitivity, and specificity. Shapley additive explanations (SHAP) values are subsequently used to identify the most influential features in the model's prediction. Logistic regression and random forest model are also developed and evaluated for comparison.

To interpret the XGBoost model, we used SHAP using shapviz(), which provides insight into the contribution of each feature to the model's prediction. Feature importance was derived from the mean absolute SHAP values.^6^

As a complement and for comparison, we also used a logistic regression model and a random forest model. Logistic regression assessed the association between features of interest and GI_bleeding by unadjusted and adjusted models. The Random Forest analysis was based on all features.

### 2.5 Adherence to recommendations

This study complies with the recommendations of Stevens et al., published in 2020 regarding the reporting of ML analyses in clinical research.^14^

### 2.6 Nomenclature of targets and ligands

Key protein targets and ligands in this article are hyperlinked to corresponding entries in http://www.guidetopharmacology.org and are permanently archived in the Concise Guide to PHARMACOLOGY 2023/24.^15^

## 3 RESULTS

### 3.1 Dataset overview

The initial dataset consisted of 2025 ICSRs. After excluding ICSRs involving less than <18 years‐old, overdoses without any ADRs and missing data, 1045 ICSRs were retained in the final dataset (Figure 2). Characteristics of the final dataset are described in Table 1.

**FIGURE 2:**
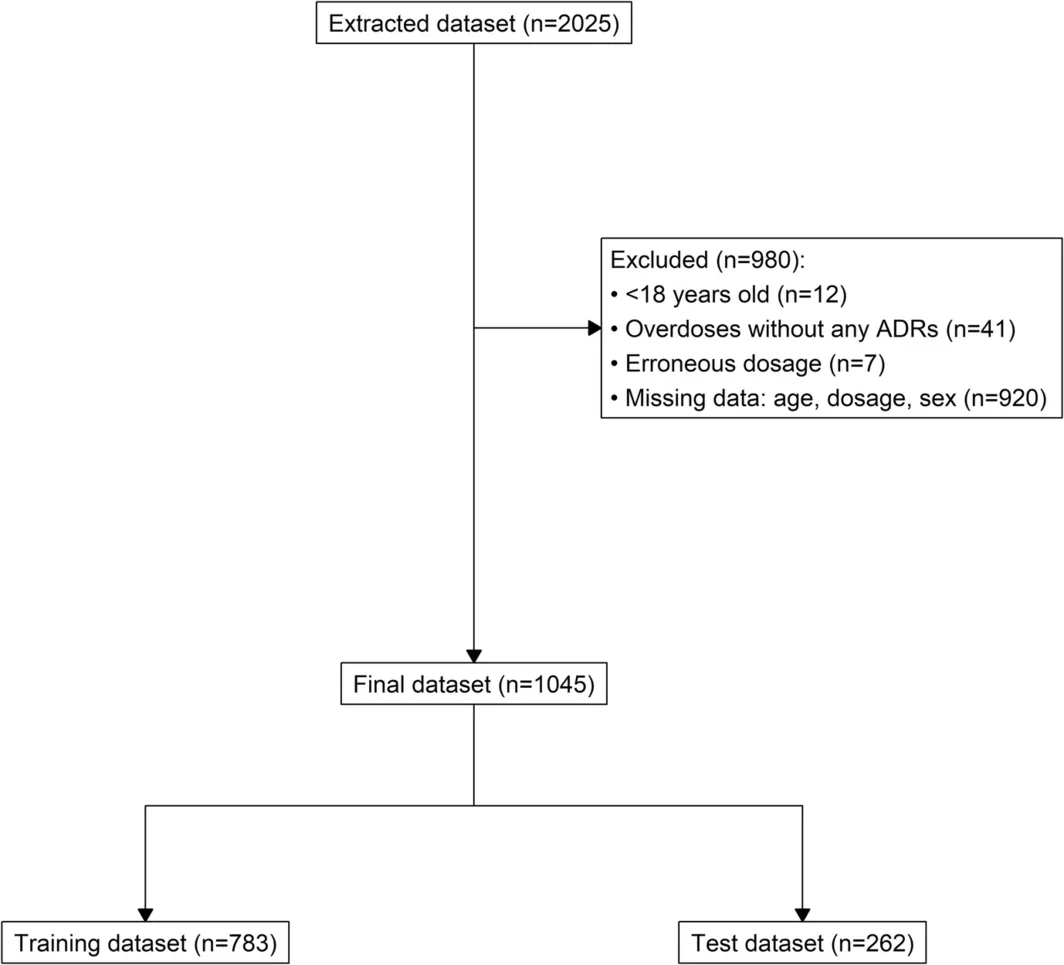
Flowchart of the selected individual case safety reports included in analysis. ADRs, adverse drug reactions.

**TABLE 1:** Individual case safety reports characteristics before data partioning.

Variable	Overall	GI_bleeding	Other adverse drug reaction
*n*	1045	591	454
Total daily dose (median [IQR])	4.00 [2.00, 5.00]	4.00 [2.00, 5.00]	4.00 [2.00, 5.00]
Age (median [IQR])	78.00 [66.00, 86.00]	82.00 [72.00, 87.50]	73.00 [55.25, 83.00]
Female sex, *n* (%)	565 (54.1)	310 (52.5)	255 (56.2)
SRI, *n* (%)	44 (4.2)	30 (5.1)	14 (3.1)
NSAID, *n* (%)	10 (1.0)	7 (1.2)	3 (0.7)
Overdoses, *n* (%)	23 (2.2)	21 (3.6)	2 (0.4)
Anticoagulant, *n* (%)	136 (13.0)	53 (9.0)	83 (18.3)
Antiplatelet, *n* (%)	136 (13.0)	101 (17.1)	35 (7.7)
Valproate, n (%)	9 (0.9)	3 (0.5)	6 (1.3)
Cephalosporin, *n* (%)	20 (1.9)	8 (1.4)	12 (2.6)
Enzyme inducer, *n* (%)	11 (1.1)	5 (0.8)	6 (1.3)
PPI, *n* (%)	202 (19.3)	111 (18.8)	91 (20.0)
GI bleeding risk factors, *n* (%)	143 (13.7)	114 (19.3)	29 (6.4)
Liver disease history, *n* (%)	35 (3.3)	26 (4.4)	9 (2.0)
Renal dysfunction history, *n* (%)	194 (18.6)	137 (23.2)	57 (12.6)
Alcohol use, *n* (%)	58 (5.6)	42 (7.1)	16 (3.5)
Smoking, *n* (%)	79 (7.6)	46 (7.8)	33 (7.3)

Abbreviations: GI, gastrointestinal; IQR, interquartile range; NSAID, nonsteroidal anti‐inflammatory drugs; PPI, proton pump inhibitors; SRI, serotonin reuptake inhibitors.

The dataset was randomly split into a training set (*n* = 783 ICSRs, 75%) and a test set (*n* = 262 ICSRs, 25%). Baseline characteristics of both datasets are presented in Table 2. The distribution of features was generally balanced between the two sets, with the exception of two features: SRI use (40 [5.1%] in training vs 4 [1.5%] in test) and alcohol use (34 [4.3%] in training vs 24 [9.2%] in test).

**TABLE 2:** Characteristics of training and validation set.

Variable	Overall	Training dataset	Test dataset
n	1045	783	262
Total daily dose (median [IQR])	4.00 [2.00, 5.00]	4.00 [2.00, 5.00]	4.00 [2.00, 5.00]
Age (median [IQR])	78.00 [66.00, 86.00]	78.00 [66.00, 86.00]	77.00 [66.00, 86.00]
Female sex, *n* (%)	565 (54.1)	430 (54.9)	135 (51.5)
SRI, *n* (%)	44 (4.2)	40 (5.1)	4 (1.5)
NSAID, n (%)	10 (1.0)	10 (1.3)	0 (0.0)
Overdoses, *n* (%)	23 (2.2)	17 (2.2)	6 (2.3)
GI bleeding, *n* (%)	591 (56.6)	443 (56.6)	148 (56.5)
Anticoagulant, *n* (%)	136 (13.0)	99 (12.6)	37 (14.1)
Antiplatelet, *n* (%)	136 (13.0)	107 (13.7)	29 (11.1)
Valproate, *n* (%)	9 (0.9)	6 (0.8)	3 (1.1)
Cephalosporin, *n* (%)	20 (1.9)	16 (2.0)	4 (1.5)
Enzyme inducer, *n* (%)	11 (1.1)	8 (1.0)	3 (1.1)
PPI, *n* (%)	202 (19.3)	151 (19.3)	51 (19.5)
GI bleeding risk factors, *n* (%)	143 (13.7)	109 (13.9)	34 (13.0)
Liver disease history, *n* (%)	35 (3.3)	23 (2.9)	12 (4.6)
Renal dysfunction history, *n* (%)	194 (18.6)	150 (19.2)	44 (16.8)
Alcohol use, *n* (%)	58 (5.6)	34 (4.3)	24 (9.2)
Smoking, *n* (%)	79 (7.6)	60 (7.7)	19 (7.3)

Abbreviations: GI, gastrointestinal; IQR: interquartile range; NSAID, nonsteroidal anti‐inflammatory drugs; PPI, proton pump inhibitors; SRI, serotonin reuptake inhibitors.

### 3.2 Machine learning model

#### 3.2.1 Extreme gradient boosting model

The importance plot used to build the final XGB model is presented in Figure 3. Age was the most important variable followed by total daily dose, GI bleeding risk factors, antiplatelet, renal dysfunction history, anticoagulant, female sex, PPI and smoking.

**FIGURE 3:**
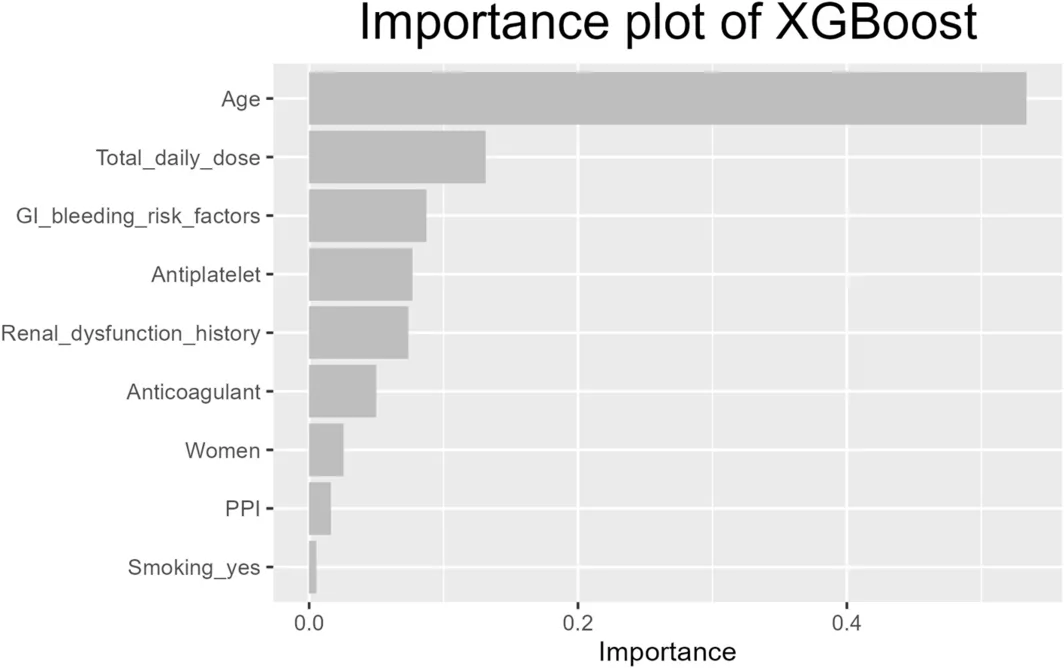
Importance plot of the XGB model.

The confusion matrix of the training dataset is available in the supplemental Table S2.

Figure 4 shows the confusion matrix of the XGB Model on the test dataset.

**FIGURE 4:**
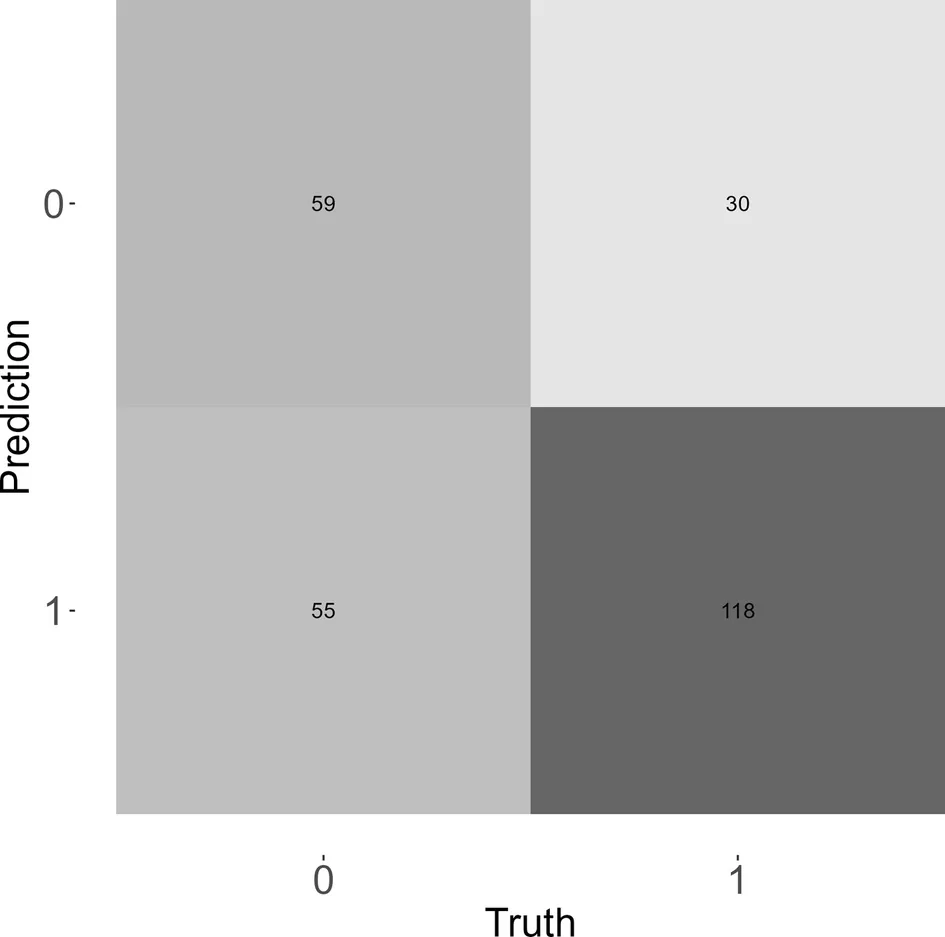
Confusion matrix of the XGB model performed on the test dataset. Values represent the number of individual case safety reports correctly and incorrectly classified for gastrointestinal bleeding. It shows the counts of true positives (*n* = 118), false positives (*n* = 55), true negatives (*n* = 59) and false negatives (*n* = 30). The colour intensity in each cell is proportional to the number of individual case safety reports classified within that category, with darker shades indicating higher counts.

XGBoost presented an accuracy of 0.68, a recall (sensitivity) of 0.80, precision of 0.68 and F1‐score of 0.74. The ROC curve is presented in Figure 5. The AUC of the ROC curve was 0.716.

**FIGURE 5:**
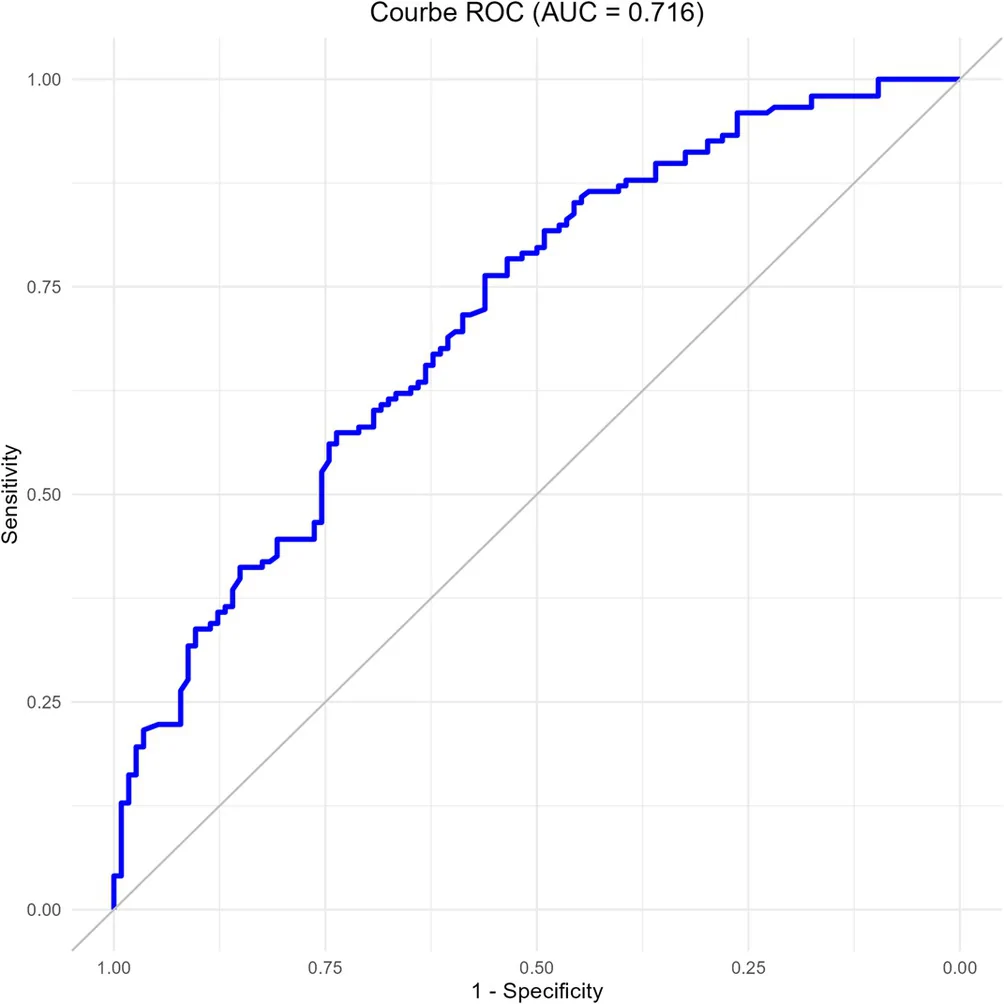
ROC curve. The area under the curve of 0.716 indicates moderate discriminative ability for predicting gastrointestinal bleeding. The diagonal line represents the performance of a random classifier.

#### 3.2.2 Shapley additive explanations

The top seven features used for the construction of the model are presented in Figure 6. They were ranked according to their mean SHAP values as follows: age, gastrointestinal bleeding risk factors, antiplatelet, renal dysfunction history, female sex (women), total daily dose, and anticoagulant. The SHAP dependence plots are presented in Figure 7 and show how the features affected the gastrointestinal bleeding prediction.

**FIGURE 6:**
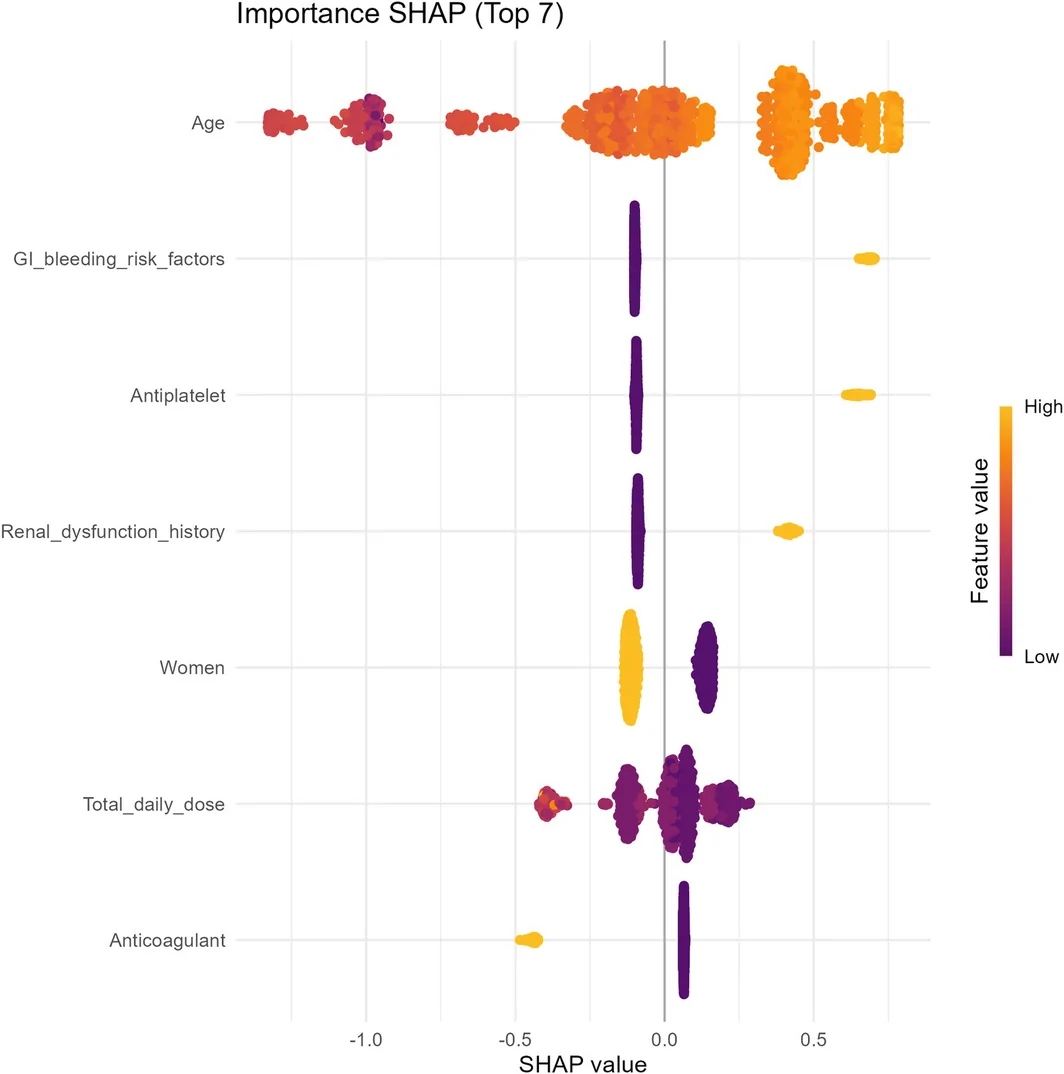
SHAP summary plot of the top 7 features. Yellow (purple) dots represent high (low) feature values. High (low) SHAP values indicate a strong positive (negative) contribution of the corresponding feature to the prediction of gastrointestinal bleeding. The order of variables in SHAP visualizations reflects their relative importance as determined by these values.

**FIGURE 7:**
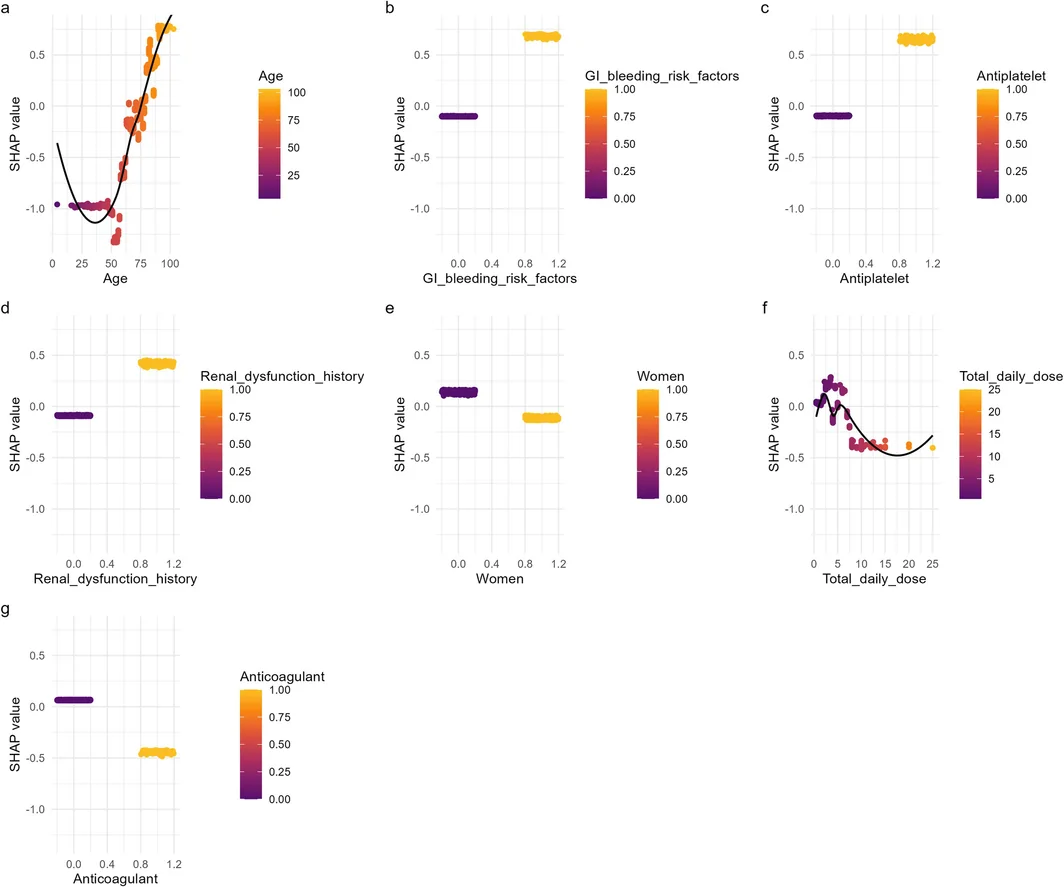
SHAP dependence plots showing the relationship between each feature and its SHAP value (i.e., contribution to the prediction of gastrointestinal bleeding). (A) age; (B) GI_bleeding_risk_factors; (C) antiplatelet; (D) renal dysfunction history; (E) women (female sex); (F) total daily dose; (G) anticoagulant. In plot (A) and (F), and a trend line, was added to emphasize the contribution of the feature to gastrointestinal bleeding reporting prediction.

#### 3.2.3 Logistic regression and random forest

Logistic regression achieved a balanced performance, with an AUC of 0.73, sensitivity of 0.80, and specificity of 0.54 (Tables S3 and S6). Random forest achieved an AUC of 0.72, sensitivity of 0.82 and specificity of 0.41 (Tables S5 and S6).

## 4 DISCUSSION

This proof‐of‐concept study demonstrates the feasibility of applying ML, specifically the XGBoost algorithm, combined with explainable artificial intelligence (SHAP) analysis on pharmacovigilance data. Our model achieved satisfactory performance for classifying gastrointestinal bleeding ICSRs, with a ROC AUC of 0.716, high sensitivity (80%) and a balanced F1‐score of 0.74. The integration of SHAP values provided transparent interpretation of the model's predictions.

ML methods, broadly defined, have mainly been used for signal detection in pharmacovigilance.^16^ In this study, we showed that an XGBoost algorithm can also be used to identify and rank predictive features of ADRs reporting from pharmacovigilance data. To our knowledge, only one previous study has applied ML (using a random forest model) to detect risk factors for bleeding associated with direct oral anticoagulants in a pharmacovigilance database (5).

Regarding model interpretability, SHAP values enhance transparency by ranking feature contributions to model predictions, without infering causality. Here, the most influential predictors identified by SHAP analysis were in order of importance: age, the presence of gastrointestinal bleeding risk factors, antiplatelet, history of renal dysfunction, female sex, total daily dose, and anticoagulant. As this study was designed as a proof‐of‐concept, the selection of features followed a primarily data‐driven approach. Notably, the most influential predictors identified by the SHAP analysis such as age, renal dysfunction and antiplatelet are well‐established risk factors for bleeding reported in cohort studies.^11^, ^12^ This concordance supports the validity of the model and suggests that such approaches could be extended to identify novel or underrecognized factors associated with ADR reporting. Two findings departed from conventional clinical interpretation: a lower total daily dose and the absence of anticoagulant. These features were associated with a higher predicted probability of having a ICSR involving GI bleeding. The dose–risk relationship was non‐linear and showed inconsistent relationship with GI bleeding. This pattern is plausibly explained by warfarin dosing practices: Warfarin dose is individualized based on INR, meaning that a higher dose does not necessarily reflect higher bleeding risk in contrast to overdose (although this latter was not a key feature in the XGBoost model).^17^ Regarding anticoagulant, most ICSRs without GI bleeding involving several anticoagulants were cases describing heparin–warfarin bridging or switch between different anticoagulants with persistent ADR or cutaneous reactions, which likely influenced the SHAP attribution. Importantly, these two features (total daily dose and anticoagulant) ranked only sixth and seventh in overall importance, indicating that they exert limited influence on the model's output. These discordants results emphazise the fact that the predictive features identified by the XGBoost model should not be interpreted as causal risk factors for GI bleeding. In this study, the XGBoost model trained on pharmacovigilance data primarily learn patterns associated with the reporting of ADRs rather than causal relationships. Consequently, the features highlighted by the model should be interpreted as factors associated with the reporting of gastrointestinal bleeding in ICSRs. Therefore, ML models performed on pharmacovigilance data require a post hoc model interpretability with a clinical and pharmacological expertise, to ensure that features impacting model predictions are in fact clinically meaningful.

Finally, for comparison, we also trained a logistic regression model and a random forest model using the same training/test split and feature set (see Tables S3–S6). Logistic regression identified other features (alcohol use and overdoses) that where not useful for the XGBoost model to predict gastrointestinal bleeding (Table S4). This discrepancy likely reflects the XGBoost model's ability to down weight variables that do not improve predictive performance despite their statistical association.

Several limitations of our study warrant acknowledgment. First, due to the nature of pharmacovigilance data, ML models can identify spurious associations or artefactual predictors due to biases in pharmacovigilance data. The selection of features was primarily data‐driven. Consequently, some relevant and/or unknown clinical features may not have been captured. Coding errors or inconsistencies in coding practices (regarding the coding of concomitant medications) between pharmacovigilance centers are possible, which may introduce bias into the results. Further, case reports without GI bleeding cannot serve as true controls, as all reports in the database are suspected ADRs. ICSRs involving warfarin do not represent the full spectrum of patients using this medication in clinical practice. Another limitation inherent to pharmacovigilance databases is underreporting. Only ADRs that are reported are captured in the database, and therefore the database does not include unreported cases. As a result, the XGBoost model cannot distinguish characteristics associated with the occurrence of an ADR from those associated with the likelihood of reporting it. In addition, characteristics related to the reporter (e.g., physician, pharmacist or patient) may influence reporting patterns and could potentially affect the predictors identified by machine learning models. Including such variables in future analyses could help better understand reporting behaviours. Pharmacovigilance databases often lack comprehensive clinical information, as key variables may be inconsistently or incompletely recorded. One potential solution to enrich data extraction is the integration of natural language processing techniques to mine nuanced clinical details from unstructured narrative text in ICSRs.

Despite these limitations, our study underscores the value of using ML in pharmacovigilance data in complement to classical approach. We found that strong predictors in our model are well‐established risk factors identified in cohort studies.^11^, ^12^ This convergence suggests that pharmacovigilance databases, when analysed with ML models and with a post hoc pharmacological validation of ML outputs, may serve not only for signal detection but also for hypothesis generation regarding novel or underrecognized risk factors.

This study was designed as a proof‐of‐concept focusing on a well‐defined drug–adverse reaction pair. We demonstrate the feasibility of using the XGBoost algorithm, a machine learning approach combined with explainable AI methods, to classify gastrointestinal bleeding cases and reporting risk factors associated with warfarin in pharmacovigilance data. This method allows to retrieve strong risk factors classically involved in bleeding with warfarin and support the utility of ML for hypothesis generation based on pharmacovigilance data. The ML approach presented here requires further refinement, and additional research is needed to improve model performance, robustness and clinical applicability. This method could potentially be applied to other drug–adverse reaction pairs in pharmacovigilance databases to explore predictors of ADR reporting.

## AUTHOR CONTRIBUTIONS

All authors contributed to the study conception and design. Material preparation, data collection and analysis were performed by Romain Barus. The first draft of the manuscript was written by Romain Barus and all authors commented on previous versions of the manuscript. All authors read and approved the final manuscript.

## CONFLICT OF INTEREST STATEMENT

The authors declare no conflicts of interest.

## Supporting information


**Table S1:** List of the drugs included for each variable.
Table S2: Confusion matrix of XGBoost on train dataset.
Table S3: Metrics performance of logistic regression on test dataset.
Table S4: Logistic regression result on the test dataset.
Table S5: Confusion matrix of random forest on test dataset.
Table S6: Comparison between the three models.

## Data Availability

Data are available upon reasonable request.
