# Supplementary material for: Applying machine learning to pharmacovigilance data: A proof‐of‐concept study
Source: Br J Clin Pharmacol. 2026 Jun 17;92(10):3503–12. doi: 10.1002/bcp.70655 (PMC13619036; doi:10.1002/bcp.70655)
Supplement: Supplementary file 1 [file BCP-92-3503-s001.docx]

Supplementary table 1: List of the drugs included for each variable

| **Variable** | **Drugs** |
| --- | --- |
| Anticoagulant | Rivaroxaban; Fluindione; Apixaban; Edoxaban; Dabigatran; Argatroban; Eptifibatide; Tirofiban; Abciximab; Defibrotide; Enoxaparin; Tinzaparin; Dalteparin; Nadroparin; Fondaparinux; Heparin; Hirudin; Acenocoumarol; Danaparoid; Desirudin; Lepirudin; Bivalirudin |
| Antiplatelet | Clopidogrel; Prasugrel; Dipyridamole; Ticlopidine; Acetylsalicylic acid; Cangrelor; Ticagrelor |
| Valproate | Valproate (Valproic acid) |
| Cephalosporin | Cefamandole; Cefbuperazone; Cefmenoxime; Cefmetazole; Cefonicid; Cefoperazone; Ceforanide; Cefotetan; Cefpiramide; Latamoxef; Cefazolin; Cefixime; Ceftriaxone |
| Enzyme inducer use | Carbamazepine; Fosphenytoin; Phenobarbital; Phenytoin; Primidone; Rufinamide; Rifabutin; Rifampicin; Efavirenz; Etravirine; Lopinavir; Nevirapine; Ritonavir; Elvitegravir; Nelfinavir; Nirmatrelvir; Enzalutamide; Apalutamide |
| PPI | Esomeprazole; Rabeprazole; Omeprazole; Lansoprazole; Pantoprazole |

Supplementary table 2: Confusion matrix of XGBoost on train dataset

|  |  | Reference | |
| --- | --- | --- | --- |
| Prediction |  | 0 | 1 |
|  | 0 | 106 | 34 |
|  | 1 | 234 | 409 |

Supplementary table 3: Metrics performance of logistic regression on test dataset

| metric | value |
| --- | --- |
| ROC AUC | 0.7306188 |
| Accuracy | 0.6908397 |
| Sensitivity | 0.8040541 |
| Specificity | 0.5438596 |
| PPV | 0.6959064 |
| NPV | 0.6813187 |

AUC-ROC: area under the receiver operating characteristic curve; NPV: negative predictive value; PPV: positive predictive value.

Supplementary table 4: Logistic regression result on the test dataset

| Variable | ROR_brut | CI95_low_brut | CI95_high_brut | p_brut | Adjusted ROR | CI95_low_adj | CI95_high_adj | p_multi |
| --- | --- | --- | --- | --- | --- | --- | --- | --- |
| **Age** | **1.0456145** | **1.0351684** | **1.056703** | **1.86e-17** | **1.0436832** | **1.0316042** | **1.0565707** | **2.26e-12** |
| SRI | 1.8441767 | 0.9450513 | 3.817600 | 8.28e-02 | 1.3667035 | 0.6519732 | 3.0141113 | 4.20e-01 |
| NSAID | 1.8035168 | 0.4973788 | 8.415874 | 3.95e-01 | 1.6926979 | 0.4255487 | 8.3373329 | 4.72e-01 |
| **GI_bleeding_risk_factors** | **2.9481894** | **1.8665226** | **4.810279** | **6.97e-06** | **2.4275313** | **1.4729566** | **4.1186981** | **6.93e-04** |
| **Renal_dysfunction_history** | **2.4774775** | **1.6834858** | **3.709256** | **6.42e-06** | **1.7067418** | **1.1096323** | **2.6608954** | **1.63e-02** |
| **Alcohol_yes** | **3.7558231** | **1.6439524** | **10.137344** | **3.69e-03** | **3.1184224** | **1.2419833** | **9.0525297** | **2.27e-02** |
| **Overdosis** | **12.7025761** | **2.5738797** | **229.833502** | **1.39e-02** | **9.6664770** | **1.8546106** | **178.1409483** | **3.10e-02** |
| Liver_disease_history | 2.2214397 | 0.9118031 | 6.213448 | 9.67e-02 | 1.7102777 | 0.6313070 | 5.1784274 | 3.10e-01 |
| **Antiplatelet** | **2.5548413** | **1.6302144** | **4.118382** | **6.83e-05** | **2.5131247** | **1.5303371** | **4.2426288** | **3.80e-04** |
| **Anticoagulant** | **0.4504950** | **0.2909205** | **0.690450** | **2.88e-04** | **0.5839325** | **0.3592721** | **0.9417558** | **2.82e-02** |
| Total_daily_dose | 0.8954111 | 0.8441231 | 0.947301 | 1.69e-04 | 0.9954440 | 0.9317050 | 1.0637082 | 8.92e-01 |
| PPI | 1.0899173 | 0.7620135 | 1.565961 | 6.39e-01 | 0.6970633 | 0.4571786 | 1.0609512 | 9.23e-02 |
| Female sex | 0.8580109 | 0.6451710 | 1.140026 | 2.91e-01 | 0.7839776 | 0.5674662 | 1.0804194 | 1.38e-01 |

ROR (reporting odds ratio) adjusted for Age, SRI (serotonin reuptake inhibitors), NSAID (non steroid anti-inflammatory agents), GI_bleeding_risk_factors (gastro intestinal), renal_dysfunction_ history, alcohol_yes, overdosis, liver_disease_history, Antiplatelet, Anticoagulant, total_daily_dose, PPI (proton pump inhibitors), Female sex.

Supplementary table 5: Confusion matrix of random forest on test dataset

|  |  | Reference | |
| --- | --- | --- | --- |
| Prediction |  | 0 | 1 |
|  | 0 | 47 | 27 |
|  | 1 | 67 | 121 |

Supplementary table 6: Comparison between the three models

| **Model** | **Accuracy** | **Sensitivity** | **Specificity** | **PPV** | **NPV** | **AUC ROC** |
| --- | --- | --- | --- | --- | --- | --- |
| **Logistic Regression** | 0.691 | 0.804 | 0.544 | 0.696 | 0.681 | 0.73 |
| **XGBoost** | 0.676 | 0.797 | 0.518 | 0.682 | 0.662 | 0.71 |
| **Random Forest** | 0.641 | 0.818 | 0.412 | 0.643 | 0.635 | 0.72 |

AUC ROC: area under the receiver operating characteristic curve; PPV: positive predictive value; NPV: negative predictive value.
